# Confined dual Lewis acid centers for selective cascade C–C coupling and deoxygenation†

**DOI:** 10.1039/d3sc06921d

**Published:** 2024-05-08

**Authors:** Houqian Li, Jifeng Pang, Wenda Hu, Vannessa Caballero, Junming Sun, Mingwu Tan, Jian Zhi Hu, Yelin Ni, Yong Wang

**Affiliations:** a The Gene & Linda Voiland School of Chemical Engineering and Bioengineering, Washington State University Pullman WA 99164 USA wenda.hu@wsu.edu junming.sun@wsu.edu; b Dalian Institute of Chemical Physics, Chinese Academy of Sciences No. 457 Zhongshan Road Dalian 116023 P.R. China; c Pacific Northwest National Laboratory Richland WA 99352 USA yong.wang@pnnl.gov; d Institute of Sustainability for Chemicals, Energy and Environment 1 Pesek Road Jurong Island 627833 Singapore

## Abstract

The selective formation of C–C bonds, coupled with effective removal of oxygen, plays a crucial role in the process of upgrading biomass-derived oxygenates into fuels and chemicals. However, co-feeding reactants with water is sometimes necessary to assist binding sites in catalytic reactions, thereby achieving desirable performance. Here, we report the design of a CeSnBeta catalyst featuring dual Lewis acidic sites for the efficient production of isobutene from acetone *via* C–C coupling followed by deoxygenation. By incorporating Ce species onto SnBeta, which was synthesized through liquid-phase grafting of dealuminated Beta, we created confined dual Lewis acidic centers within Beta zeolites. The cooperative action of Ce species and framework Sn sites within this confined environment enabled selective catalysis of the acetone-to-isobutene cascade reactions, showcasing enhanced stability even without the presence of water.

## Abstract 2

Dual Lewis acidic centers, CeO_*x*_ and framework Sn, confined in Beta zeolite, catalyze the acetone C–C coupling followed by deoxygenation to isobutene, achieving a desirable product distribution and stability in the absence of water.

## Graphical abstract

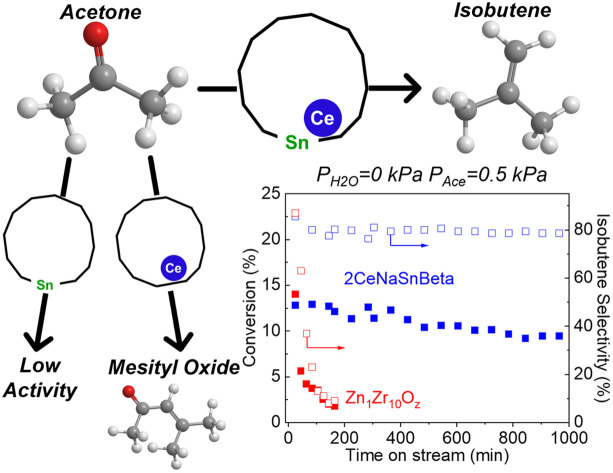

Inspired by natural enzymes with confined binding sites and micro environments, heterogeneous enzyme mimics have been extensively explored.^1^ Zeolites, with well-defined structure, are ideal supports to host active centers with confined environments.^2–4^ However, the catalytic performance and structure–function relationship of isolated metal cations surrounded by inorganic scaffolds created as uniform Lewis acid sites or redox partners remain to be further investigated.^5^ The areas to which these materials can potentially be applied include biomass conversion, vehicle emission control, and C_1_ conversion, where catalysts with densely packed or isolated metal cations are widely used.^6–8^

Selective C–C coupling and oxygen elimination play a crucial role in biomass upgrading. For example, biomass-derived ethanol/acetone can be used to produce isobutene, one of the key building blocks in the chemical industry, *via* cascade C–C coupling and deoxygenation reactions catalyzed by Lewis acidic centers in the presence of water.^6,9,10^ The synergy between water and balanced Lewis acid–base pairs (*e.g.*, Zn–O–Zr) generates an appropriate local environment, enabling the production of isobutene from acetone (Scheme 1) with desirable catalyst stability and selectivity.^11^ This reaction can also be catalyzed by Brønsted acidic zeolites, however, accompanied with catalyst deactivation even when water is present.^12^ Among these studies, co-fed water is critical to mitigate the severe deactivation of catalysts utilized in the acetone-to-isobutene conversion.^9,11,13^ Due to the high temperature (473–723 K) needed for this reaction, necessitating significant energy for water heating and potential post-treatment, it would be desirable to conduct this reaction without co-fed water. However, no known catalytic materials selectively catalyze this reaction without water, and stabilizing the isobutene precursor (*e.g.*, diacetone alcohol-derived intermediates) without water remains an unknow challenge.

**Scheme 1:**
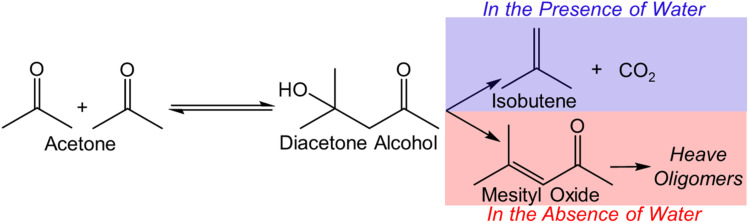
Proposed reaction pathway for the acetone conversion over Lewis acid-based pairs on metal oxides. The surface primary reaction can be tuned by adjusting the local environment through cofeeding or not cofeeding water. The formation of mesityl oxide could lead to the generation of heavy oligomers which further deactivates the metal oxide catalysts.

Herein, we report a CeSnBeta catalyst featuring confined dual Lewis acidic centers (Ce and Sn cations), which exhibits remarkable selectivity in converting acetone to isobutene, achieving nearly 100% theoretical selectivity to isobutene. Notably, this catalyst demonstrates enhanced stability, showing negligible activity decline over 17 h time-on-stream at conversions below 15%, even in the absence of co-fed water. Our studies reveal that the confined dual Lewis acidic centers (Ce and Sn cations) likely provide unique binding sites and local environment for the stabilization of key intermediates, facilitating the stable and selective production of isobutene from acetone.

Commercial Beta zeolites were dealuminated for the synthesis of a series of *x*CeSnBeta and *x*CeSnNaBeta (*x* represents weight percentage of Ce) catalysts. Details of the synthesis and related experiments can be found in ESI.† The obtained catalysts, with similar surface area and pore volume (Table S1†), were evaluated in acetone-to-isobutene reaction *via* cascade C–C coupling, self-deoxygenation, and C–C bond cleavage reactions (Scheme 1),^11^ and the results are shown in Fig. 1. Over Beta-deAl, 73% isobutene was obtained at 4% acetone conversion, suggesting that acid sites on Beta-deAl show limited activity for this cascade reaction. We postulate that the acid sites, either originating from the trace amount of framework Al (Brønsted acidic, Table S2†) or silanol nests (Brønsted or Lewis acidic), catalyze the cascade reactions.^14,15^ This is supported by the extreme low activity (<1% conversion) observed on Na^+^ titrated Beta-deAl (NaBeta-deAl in Fig. 1). Compared with Beta-deAl, incorporation of Sn into Beta zeolite increased the acetone conversion slightly to 7% with a marginal increase of isobutene selectivity, likely due to the promoted C–C coupling of ketones by framework Lewis acidic Sn sites.^3^ However, the observed inactivity in the NaSnBeta catalyst suggests that Sn species, lacking protons that could be titrated by Na^+^, are incapable of efficiently catalyzing one or multiple reaction steps in these cascade reactions, such as enolization, C–C coupling, decomposition, and ketonization.^11^

**Fig. 1:**
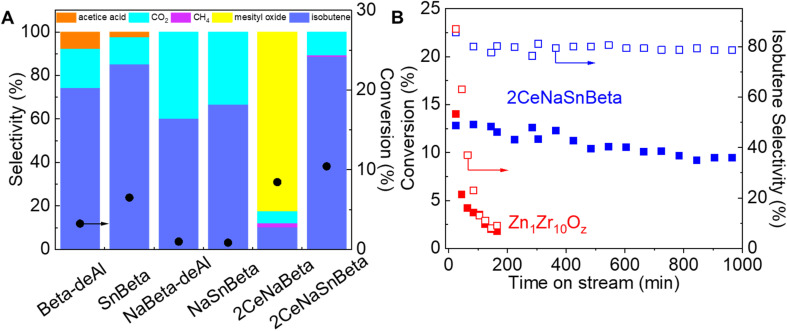
(A) Performance of Beta-deAl, SnBeta, NaBeta-deAl, NaSnBeta, 2CeNaBeta, and 2CeNaSnBeta for acetone conversion (*P*_Acetone_ = 0.5 kPa, 673 K, space velocity = 0.23 gAce/gcat/hr, averaged 40–120 min time on stream results, carbon balance >85%). (B) Stability of 2CeNaSnBeta (*P*_Acetone_ = 0.5 kPa, 673 K) and of Zn_1_Zr_10_O_*z*_ (*P*_Acetone_ = 0.5 kPa, 673 K) for acetone conversion. The product distribution over 2CeNaSnBeta during the stability test remains the same as shown in (A).

Basic or amphoteric metal oxides (*e.g.*, ZnO, CeO_2_) have been reported to accelerate enolization of acetone,^16^ C–C coupling followed by decomposition of generated dimers,^17^ and ketonization reactions.^18^ To improve the performance of SnBeta catalysts, Ce species were introduced and evaluated under the same conditions. Interestingly, the introduction of Ce species into NaBeta-deAl initiates acetone aldolization as evidenced by the increased acetone conversion on the 2CeNaBeta (Fig. 1A). However, a high selectivity to mesityl oxide was also observed on 2CeNaBeta, accompanied by catalyst deactivation, suggesting that Ce species in Beta-deAl cannot efficiently catalyze the C–C cleavage of acetone dimers (*e.g.*, diacetone alcohol, mesityl oxide). In contrast, desirable product distribution was achieved over 2CeNaSnBeta (Fig. 1B) indicates that framework Sn sites, along with introduced Ce species, play a significant role in mediating reaction pathway. Zn_*x*_Zr_*y*_O_*z*_ mixed metal oxide was the first catalyst reported being able to catalyze this reaction achieving great stability and theoretical selectivity in the presence of water.^9^ However, severe deactivation accompanied with decreased selectivity to isobutene were observed without cofed water (Fig. 1B). Compared with Zn_*x*_Zr_*y*_O_*z*_, 2CeNaSnBeta shows drastically enhanced stability as well as the selectivity to isobutene even in the absence of water. The reusability test of this material (Fig. S1†) suggests that the catalytic active sites will not be affected by oxygen treatment. This treatment could be employed to regenerate the catalyst by burning out the coke on the surface.

To unravel the site requirements of this reaction on the obtained materials, the chemical and physical properties of confined dual Lewis acidic Ce and Sn centers were examined using XRD, ^1^H-SSNMR, diffuse reflectance (DR) UV-Vis (Ultraviolet-Visible Spectroscopy), and Diffuse Reflectance Infrared Fourier Transform Spectroscopy (DRIFTS)-pyridine. By employing a well-established dealumination procedure,^14^ framework Al species were extensively removed to yield Beta-deAl with a Si/Al = 600, as confirmed by ^27^Al-solid state nuclear magnetic resonance (SSNMR) and inductively coupled plasma (ICP) analysis (Fig. S2 and Table S2†, respectively). The introduction of Sn was confirmed by ICP, achieving 0.3 wt% Sn. X-ray diffraction (XRD) of all the materials (Fig. S3†) exhibit typical Beta patterns, indicating the Beta framework was maintained after the dealumination process as well as post-synthetic procedures. XRD patterns ascribed to CeO_2_ were not observed until CeO_2_ loading is higher than 5%, suggesting a highly dispersed Ce species on SnBeta at low Ce loading (*e.g.*, 2 wt%), which was also confirmed by scanning transmission electron microscopy results (Fig. S3†). DRUV-Vis was employed to characterize Beta-deAl and SnBeta samples. As shown in Fig. 2A, peaks at 220 and 315 nm in the Beta-deAl spectrum are attributed to the charge transfer between framework oxygen anions and Si or trace amount of Al.^19^ The presence of these two peaks in SnBeta spectrum is consistent with the low loading (0.3 wt%) of Sn introduced by liquid phase grafting method. Upon Sn addition, the peak at <220 nm belonging to framework tetrahedrally coordinated Sn^IV^ appeared, while the peak at 270 nm was not observed suggesting the absence of extra-framework SnO_2_ species, consistent with previous reports.^20,21^Fig. 2B shows the DRUV-Vis spectra of *x*CeSnBeta materials. At low Ce loadings (*i.e.*, 0.4CeSnBeta and 2CeSnBeta), the absorption peak centered at 260 nm was observed, which is ascribed to Ce^III^-oxygen charge transfer in small cerium oxide particles (*i.e.*, <1.5 nm).^22^ As the Ce loading further increases (10CeSnBeta), this peak shifts to a higher wavelength, indicating an increased ratio of Ce^IV^-oxygen and an increased domain size of Ce species.^22^ DRIFTS-pyridine was further performed to study the surface acidity of the catalysts, as shown in Fig. 2C. In all samples, peaks at 1448 and 1596 cm^−1^ belong to the 8a and 19b band pyridine adsorption *via* hydrogen bond while the shoulder peak at 1575 cm^−1^ is likely resulted by physisorbed pyridine.^23^ Although Brønsted acidic centers have been proposed on SnBeta due to silanols adjacent to framework Sn site^24^ or residual Al,^21^ pyridine adsorbed on Brønsted acidic site at 1540 cm^−1^ was not detected on all the samples, likely due to the low content of Al (Table S2†). The shoulder peak at 1615 cm^−1^ in the spectra of SnBeta and 2CeSnBeta is assigned to pyridine bound to Lewis acidic Sn sites.^23^ After the introduction of Ce species, a new peak at 1486 cm^−1^ assigned to oxidized pyridine adsorbed on Lewis acid sites appears, which has been previously observed on CeO_*x*_.^23^

**Fig. 2:**
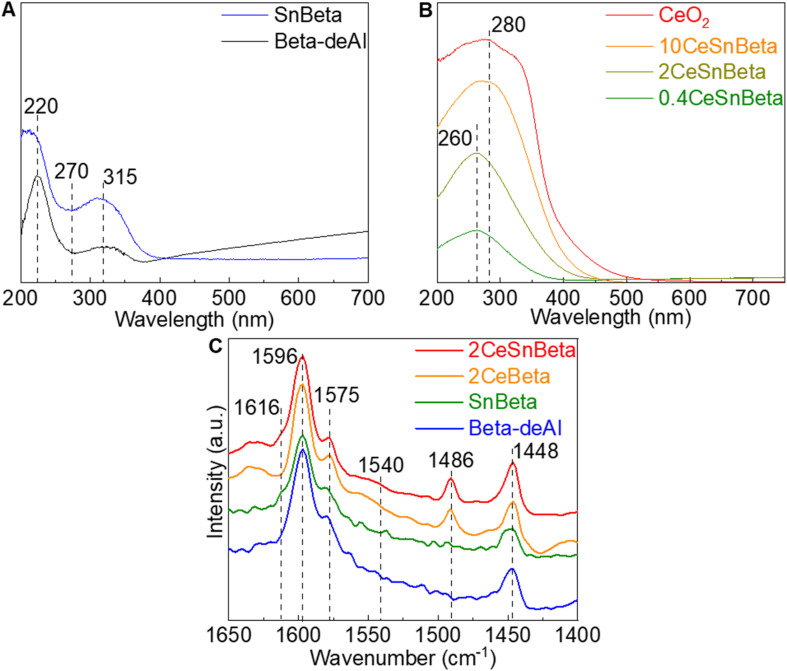
(A) DRUV-Vis of Beta-deAl and Sn Beta. (B) DRUV-Vis of CeSnBeta. (C) DRIFTS-pyridine spectra of Beta-deAl, SnBeta, 2CeBeta, and 2CeSnBeta catalysts at 423 K.

Fig. 3 depicts the ^1^H-SSNMR spectra of the synthesized catalysts. No protons associated with Brønsted acid sites were detected (4 ppm),^25^ possibly due to concentration of the acid sites below the detection limit of ^1^H-SSNMR. Indeed, the signal becomes too broad to be detected if most of Brønsted protons are removed.^25^ The sharp peak at 2 ppm, the shoulder peak at 1.7 ppm, and the broad peak at 2.5–4 ppm belong to protons in silanol nests, terminal silanols, and hydrogen bond interaction of silanols in nest, respectively.^14^ Compared with Beta-deAl spectrum, drastic decreased peak intensity at 2 ppm in SnBeta (Fig. 3A) and 2CeBeta spectra (Fig. 4A) indicates that silanol nests are perturbed by the introduction of Sn or Ce. Semi-quantitative analysis based on the integral peak area (Table S3†) reveals that about 10–20% of the silanol nests are occupied by introduced Sn. The further introduction of Ce (Fig. 3A, 2CeSnBeta) does not significantly change these two peaks, indicating that Ce species, at a loading of 2 wt%, are uniformly dispersed in the zeolite. Given the larger radius of Ce (III or IV, >100 pm) than that of Al (III, 53 pm), we postulate that majority of Ce species would not be incorporated into the framework of Beta.

**Fig. 3:**
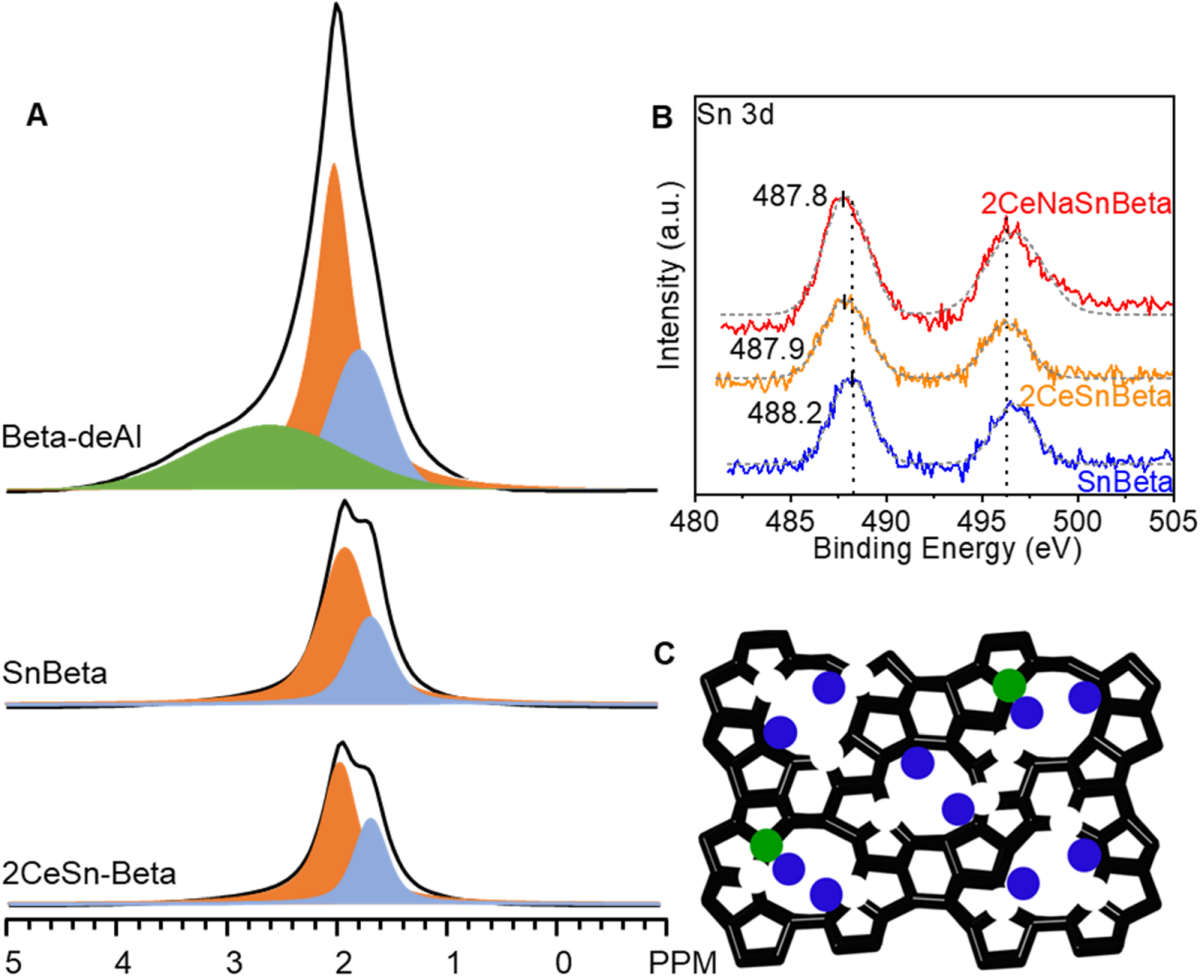
(A) ^1^H-SSNMR spectra of Beta-deAl, SnBeta, and 2CeSnBeta. (B) XPS of SnBeta, 2CeSnBeta, and 2CeNaSnBeta. (C) Proposed structure of 2CeSnBeta. Green and blue circles represent framework Sn sites and Ce species. The size of the circles does not reflect the ratio of the actual size of the species.

**Fig. 4:**
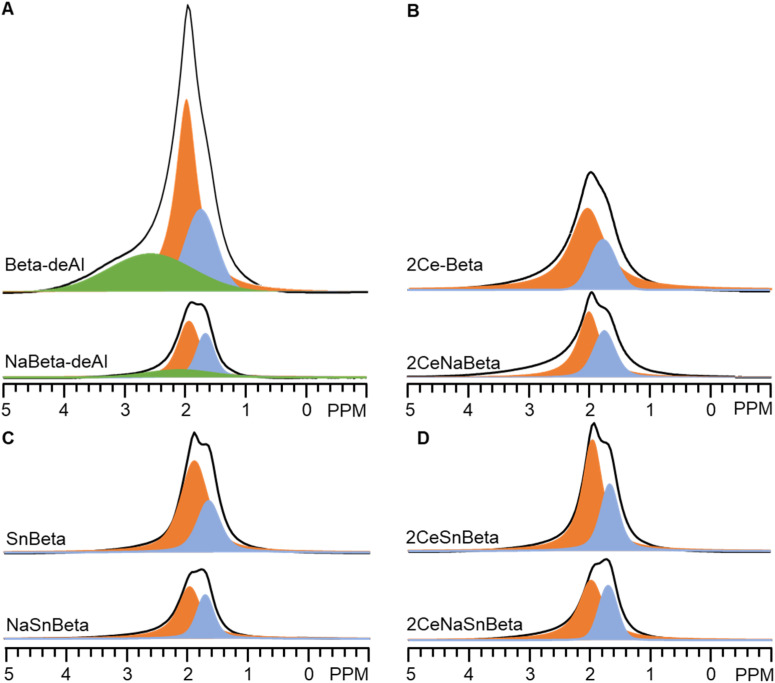
(A) ^1^H-SSNMR spectra of Beta-deAl and NaBeta-deAl. (B) ^1^H-SSNMR spectra of 2Ce-Beta and 2CeNaBeta. (C) ^1^H-SSNMR spectra of SnBeta and NaSnBeta. (D) ^1^H-SSNMR spectra of 2CeSnBeta and 2CeNaSnBeta.

It has been reported that closed (Sn–(OSi≡)_4_), hydrolyzed-open ((HO)–Sn–(OSi≡)_3_)⋯HO–Si), and defect-open ((HO)–Sn–(OSi≡)_3_) Sn sites exist on SnBeta materials which could be quantified by varied approaches.^24,26,27^ Based on the CD_3_CN-DRIFTS results (Fig. S4†), we postulate that both defect open and closed Sn sites exist on the SnBeta synthesized by liquid-phase grafting method.^28^ To understand the oxidation states of the Sn and Ce species, X-ray photoelectron spectroscopy (XPS) was performed over SnBeta, 2CeSnBeta, and 2CeNaSnBeta. In Fig. 3B, the Sn 3d peak at ∼488 eV indicates that oxidation states of Sn in these samples are Sn^4+^. It is noteworth that binding energy of Sn shifted from 488.2 eV in SnBeta to 487.9 eV in 2CeSnBeta and 2CeNaSnBeta, which indicates the role of Ce is donating electron to d-orbital of Sn. As for Ce species, the similar ratio of Ce^4+^ to Ce^3+^ in 2CeSnBeta and 2CeNaSnBeta in Ce 3d XPS spectra (Fig. S5†) suggests that Ce is interacting with Sn instead of Na^+^. Based on these reports and the obtained characterization results, the structure of CeSnBeta catalysts was proposed and depicted in Fig. 3C.

As aforementioned, acidic sites, including Lewis acidic sites (silanol nests^14^), Brønsted acidic sites (silanol nests,^15^ hydroxyls due to residual Al,^21^ or silanols adjacent to framework Sn site^24^), or both sites,^21^ may exist on Beta-deAl and SnBeta. To verify the roles of acidic protons, Na^+^ titrated catalysts, *i.e.*, NaBeta-deAl, NaSnBeta, CeNaBeta, and 2CeNaSnBeta, were also prepared and characterized by ^1^H-SSNMR as shown in Fig. 4. Compared with those without Na^+^ titration, the intensity of the peaks at 1.7, 2, and 2.5–4 ppm decreased drastically on the corresponding Na^+^ titrated samples, suggesting that majority of the protons of the hydroxyls were replaced by Na^+^ species. Per NMR semi-quantification (Table S3†), the amount of –OH on the NaBeta-deAl and NaSnBeta decreased by about 67% and 64% relative to that of Beta-deAl. A higher intensity of 1.7 and 2 ppm peaks in 2CeSnBeta spectrum relative to those on NaBeta-deAl or NaSnBeta suggests that Sn and Ce only titrates a subset of silanols while Na^+^ titrates the rest of titratable hydroxyls. Based on the ^1^H-SSNMR measurements, we conclude that acidic protons are titrated by Na^+^ over the synthesized catalysts which results in neutralized silanols on surface.

In the mechanistic study of acetone-to-isobutene reaction over mixed metal oxides (*i.e.*, Zn_*x*_Zr_*y*_O_*z*_), we demonstrated that the local environment plays a crucial role in mediating the further reaction of diacetone alcohol, the product of acetone C–C coupling.^11^ Particularly, the appropriate local environment can hinder the formation of mesityl oxide *via* dehydration and facilitate the production of isobutene *via* decomposition of diacetone alcohol. In Fig. 1, compared with their parent materials, the drastically decreased activity on the NaBeta-deAl and NaSnBeta indicates that Beta-deAl or SnBeta is unable to catalyze the cascade reactions without the active hydroxyls (*i.e.*, charge-compensating protons, silanol nests, or the silanols are titrated). Based on the eqn (3) CH_3_COCH_3_ → 2 i-C_4_H_8_ + CO_2_, the theoretical carbon selectivity to isobutene from acetone is 88.9%. On 2CeNaBeta, 10% theoretical selectivity to isobutene, accompanied by high selectivity (80%) to mesityl oxide, indicates that the transition state for isobutene formation is not favorable. In contrast, on 2CeNaSnBeta, >93% theoretical selectivity to isobutene is achieved exclusively due to the cooperative action of both the Ce and framework Sn sites. Considering the similar effects of Na^+^ interacting with the Ce species, the presence of dual Lewis acidic centers (Sn and Ce) on 2CeNaSnBeta clearly provides unique binding sites. These binding sites play a crucial role in forming and stabilizing the transition state during the production of isobutene through self-deoxygenation and C–C cleavage of the formed oligomers. Mesityl oxide was also employed as a reactant to study its conversion over 2CeNaSnBeta under this condition (Fig. S6,†*P*_Mesityl oxide_ = 0.07 kPa, 673 K, without water). Although isobutene was observed, it is unclear whether it is generated directly from mesityl oxide or from acetone/diacetone alcohol, which is the product of the mesityl oxide reverse reaction. Additionally, severe deactivation (>30% activity loss in 60 min) implies that the formation of isobutene from mesityl oxide is not the dominant reaction pathway in acetone-to-isobutene reaction over this material. The detailed reaction pathway on this catalyst remains to be further investigated. A higher conversion rate (about a factor of 2) was observed on 2CeSnBeta (Fig. S7†) than that on 2CeNaSnBeta, suggesting that active hydroxyls also contribute additionally to the activities, or unmodified Sn sites with proximal silanol are more active.^15,29^ To unambiguously clarify the roles of confined dual Lewis acidic centers in the cascade reactions, in this work, we focus on Na^+^-titrated samples to exclude the contributions by hydroxyl groups. It is noteworthy that CeO_2_ alone does not contribute to the enhanced activity since separate experiments show dominant formation of the undesirable product distribution (selectivity to mesityl oxide >50%) and marginal enhancement of acetone conversion over CeO_2_, 2%CeO_*x*_0.4%SnO_*x*_/SiO_2_, or 2%CeO_*x*_/SiO_2_ catalysts (Fig. S7†). The evaluation of cooperative Ce and framework Sn in zeolites with other topological structures (Fig. S7†) resulted in an undesirable product distribution. Different from the Zn_*x*_Zr_*y*_O_*z*_ mixed metal oxides that requires cofed water to modify the local environment and prevent catalyst deactivation, CeSnBeta likely offers a confined environment in which specific pore geometry combined with the dual Lewis acidic metal centers promotes the direct conversion of acetone to isobutene even in the absence of cofed water (Fig. 1B).

In summary, we report a novel durable catalyst for the conversion of acetone to isobutene, even in the absence of cofed water, which has not been reported previously. The CeSnNaBeta catalyst contains isolated dual Lewis acid centers confined within the Beta zeolite framework, enabling robust activity in cascade reactions involving C–C coupling, C–C cleavage, and ketonization. This study highlights how catalysts with dual metal cation centers can be designed to provide unique binding sites and a favorable local environment for important biomass upgrading reactions, such as C–C coupling followed by self-deoxygenation. The characterization of active sites confirms that isolated Ce species in Beta, without active hydroxyls, can selectively catalyze the production of isobutene from acetone oligomers in the presence of framework Sn species. In the absence of Sn species (2CeNaBeta), dehydration of diacetone alcohol occurs, yielding mesityl oxide. Although further investigations may be needed to elucidate other specific site requirements such as the domain size of Ce species and the effects of void size, this work represents a new strategy for designing catalysts with dual-metal cation centers showing superior performance.‡‡The authors have cited additional references within the ESI regarding the synthesis of SnMFI and SnSBA-15, as well as the procedure for processing NMR spectra.^30,31^

## Data availability

All data related to this article have been included in the ESI.†

## Author contributions

H. Li: conceptualization; data curation; formal analysis; investigation; methodology; visualization; roles/writing – original draft; writing – review & editing. J. Pang: methodology; roles/writing – review & editing. W. Hu: data curation; investigation; writing – review & editing. V. Caballero: data curation. J. Sun: conceptualization; writing - review & editing. M. Tan: data curation; writing – review & editing. J. Z. Hu: methodology. Y. Ni: data curation. Y. Wang: conceptualization; supervision; funding acquisition; project administration; resources; writing – review & editing.

## Conflicts of interest

The authors declare no competing financial interest.

## Supplementary Material

SC-015-D3SC06921D-s001
