# Supplementary material for: Confined dual Lewis acid centers for selective cascade C–C coupling and deoxygenation
Source: Chem Sci. 2024 May 8;15(21):8031–7. doi: 10.1039/d3sc06921d (PMC11134334; doi:10.1039/d3sc06921d)
Supplement: SC-015-D3SC06921D-s001 [file SC-015-D3SC06921D-s001.pdf]

Electronic Supplementary Material (ESI) for Chemical Science.
This journal is © The Royal Society of Chemistry 2024

Supporting Information

Confined Dual Lewis Acid Centers for Selective Cascade C-C Coupling and Deoxygenation

Houqian Li [a], Jifeng Pang [b], Wenda Hu [a, c], Vannessa Caballero [a], Junming Sun [a*], Mingwu Tan[d], Jian Zhi Hu [c], Yelin Ni [c], Yong Wang [a, c*]*

[a] The Gene & Linda Voiland School of Chemical Engineering and Bioengineering, Washington State University, Pullman, WA 99164, United States

[b] Dalian Institute of Chemical Physics, Chinese Academy of Sciences, No. 457 Zhongshan Road, Dalian 116023, P.R. China

[c] Pacific Northwest National Laboratory, Richland, WA 99352, United States

[d] Institute of Sustainability for Chemicals, Energy and Environment, 1 Pesek Road Jurong Island Singapore, 627833

*Corresponding authors: wenda.hu@wsu.edu, junming.sun@wsu.edu, yong.wang@pnnl.gov

Materials

Acetone (J.T. Baker, 99.8%), commercial BEA zeolite (Zeolyst, CP814E*, SiO_2/Al_2O_3 molar ratio=25), nitric acid (Sigma-Aldrich, ACS reagent, 70%), tin(IV) chloride pentahydrate (Sigma-Aldrich, $SnCl_4\,5H_2O$, 98%), sodium nitrate ($NaNO_3$, Sigma-Aldrich, ≥99.0%), fumed silica (Sigma-Aldrich), cerium(IV) oxide (Sigma-Aldrich, CeO_2, ≥99.0%) and cerium(III) nitrate hexahydrate (Sigma-Aldrich, $Ce(NO_3)_36H_2O$) were used as purchased.

The ammonium form of the BEA samples was transformed to the proton form (H-BEA) by calcination at 823 K for 24 h. The dealuminated BEA (Beta-deAl) was synthesized via dealuminating H-BEA by stirring it in a 13 M aqueous nitric acid solution at 373 K for 12 h (100 mL g^{-1} zeolite). The powder was then filtered, rinsed thoroughly with water (until pH≈7) and dried at 333 K.

SnBeta was synthesized by a liquid phase grafting method.[14] Before the grafting of tin, the powder was activated overnight at 423 K to remove physisorbed water. Next, the sample was suspended in isopropanol (50 mL per gram) and 27 mmol of $SnCl_4{\cdot}5H_2O$ per gram of support was added. The solution was refluxed under N_2 for 7 hours and afterwards filtered, rinsed first with isopropanol then with ethanol (until pH≈7) and dried at 333 K. The calcination procedure was as follows: ramping 3 K/min to 473 K, dwell for 6 hours, ramping 3 K/min to 823 K and dwell for 6 hours.

The sodium titrated NaBeta-deAl and NaSnBeta was obtained via an ion exchange method. The Beta-deAl or SnBeta was suspended in a 1 M $NaNO_3$ solution (water, 50 mL g^{-1} material) and the mixture was vigorously stirred at 353 K overnight. The sample was recovered by filtration followed by another time of ion exchange using the same procedure. The materials were then washed thoroughly with water (e.g., 1.5 L per gram material), filtered, and calcined at 823 K for 5 hours (5 K/min).

Cerium was introduced by a wetness impregnation method. A $Ce(NO_3)_3$ solution was added to Beta-deAl, SnBeta, NaBeta-deAl, and NaSnBeta to achieve certain weight percentage of Ce (x%) and named as xCeBeta-deAl, xCeSnBeta, xCeNaBeta-deAl, and xCeNaSnBeta. The mixture was then dried at room temperature overnight, at 378 K for 4 hours, and calcined at 823 K for 5 hours (5 K/min).

2%Ce/SiO_2, and 2%0.4%Sn/SiO_2 were synthesized by wetness impregnation method described above. SnMFI and SnSBA-15 were synthesized according to previous reports,[30] the same procedure described above was used for the incorporation of Ce.

Catalyst Characterization

Nitrogen Sorption. Surface area and pore volume of the catalysts were determined via nitrogen sorption experiments carried out on a Micromeritics TriStar II 3020 physisorption analyzer. Catalyst samples were degassed at 350 °C for 3 h under vacuum before experiments were carried out at a temperature of −196 °C.

XRD Measurements. X-ray diffraction (XRD) patterns were collected with a Rigaku Miniflex 600 apparatus equipped with a Cu Kα radiation (λ = 0.15406 nm). Bragg's angles were between 10° and 90° with a scanning rate at 1.4 deg/min. Inductively Coupled Plasma (ICP) was performed in Galbraith Laboratories.

Microscopy Imaging. Transmission electron microscopy imaging was performed on a FEI Titan 80–300 operated at 300 kV. The images were acquired with a High Angle Annular Dark Field Detector in Scanning Transmission Electron mode. The semi-convergence angle was set to 17.8 mrad, and the inner collection angle was set above 54 mrad. Compositional analysis was performed with a JEOL ARM 200 operated at 200 kV. The microscope houses a high-collection angle Silicon Drift Detector SDD (100 mm2). The samples were prepared by dispersing a dry powder on a lacey-carbon coated 200 mesh Cu grids.

DR UV-Vis Measurements. Diffuse Reflectance Ultraviolet–Visible Spectroscopy (DR UV-Vis) reflectance from 200 nm to 800 nm was measured on an Agilent Cary 5000 UV-Vis-NIR spectrophotometer with diffuse reflection accessory. A powder cell containing a thin layer of sample was mounted to the reflectance port. Reflectance (R) of a sample is made relative to the reference material, barium sulfate ($BaSO_4$), which was used to establish the 100% R baseline. The 0% R baseline was obtained by removing the powder cell from reflectance port and allowing the light to be trapped by the sample compartment. The absorbance (A) was calculated using A=-logR.

DRIFTS Measurements. Diffuse Reflectance Infrared Fourier Transform Spectroscopy (DRIFTS) - Pyridine and CD_3CN were performed with a Bruker Tenser 27 FTIR. About 20 mg catalyst was loaded into the Praying Mantis in situ cell. The sample

was first treated in 5% Ar/He (50 SCCM) at 723 K for 30 mins to remove surface impurities. The background was taken using treated sample at 323 K. Pyridine and CD_3CN was then introduced into the cell via a bubbler at room temperature. After 10 mins adsorption, 5% Ar/He was flowed (50 SCCM) for 30 mins at the temperature where background was taken to remove surface physisorbed species. The sample was then ramped to a certain desorption temperature at 10 K/min. After 30 mins purging at each desorption temperature, the catalyst was cooled down to 323 K where spectra were scanned.

^{1}H MAS NMR Measurements. In situ ^{1}H MAS (magic angle spinning) NMR (nuclear magnetic resonance) spectra were collected with a 300 MHz Varian Inova NMR spectrometer operating at a ^{1}H Larmor frequency of 299.97 MHz. All catalysts were pre-treated in a glass tube under flowing Ar at 400 °C for 2 h, then sealed and moved into a glovebox. A home-made in situ NMR rotor was used to hold the samples. Samples sealed in the rotor were spun at the magic angle at a spinning rate of 4,000 Hz in a commercial 7.5 mm ceramic probe. A single-pulse width of 4 μs, a recycle delay time of 5 s, and an acquisition time of 0.1 s were used to collect 1000 number of scans per spectrum. Quantitative fit of the SP MAS NMR spectra was conducted in NUTS. Specifically, baseline fitting was employed to eliminate the background signal. Deconvolution of peaks was conducted using mixed Gaussian/Lorentzian line shapes followed by peaks fitting and integration.

^{27}Al MAS NMR Measurements. Quantitative ^{27}Al single pulse MAS NMR experiments were conducted on a Varian Inova 850 MHz NMR spectrometer operating at a magnetic field of 19.9 T that is equipped with a 3.2 mm pencil-type commercial MAS probe. All the spectra were obtained at 221.41 MHz using a single pulse (SP) sequence at a pulse angle of 45 deg (0.5 μs) and a recycle delay of 1 s. The samples are all fully hydrated because all the sample were exposed to ambient conditions for months before the measurements. The spectra were accumulated between 5000 and 100000 scans at a sample spinning rate of 19 kHz. All spectra are externally referenced to a 1 M $Al(NO_3)_3$ aqueous solution (i.e., the 0 ppm position).[31]

XPS Measurements. X-ray photoelectron spectroscopy (XPS) analysis was conducted on a ThermoFisher ESCALAB250 instrument (Physical Electronics) with Al Kα

irradiation (1486.6 eV) at the pressure of 1.3×10^{-9} mbar. The binding energy values were referenced to the C 1s at 284.4 eV.

Catalytic Activity and Rate Measurements

The conversion of acetone was performed in a fixed-bed stainless steel reactor (i.d. 5 mm). A thermocouple was placed in the middle of the catalyst bed to monitor the temperature. A certain amount (10-100 mg) of catalyst diluted with SiC (500 mg in total) was loaded into the reactor. The catalyst was pretreated at 723 K (ramping in N_2 50 SCCM, ramping rate: 10 K/min) in N_2 for 0.5 h (50 SCCM) then decreased to reaction temperature (673 K). Acetone was fed to the evaporator via a syringe pump and carried into the reactor by N_2. The temperature of evaporator was 393 K to enable the evaporation of acetone. Shimadzu GC-2014 gas chromatograph equipped with HP-Plot Q column (30m, 0.53mm, 40 μm), flame ionization detector (FID) and thermal conductivity detector (TCD) was used to analyze all products. Acetone conversion (X), product selectivity (S_i), and carbon balance (CB) were calculated as follows:

Conversion (X) = $(mol_{acetone\text{-}in} - mol_{acetone\text{-}out}) / mol_{acetone\text{-}in}$

Selectivity species-i (Si) = $(mol_{species\text{-}i} \cdot \alpha i) / (mol_{converted\ acetone} \cdot 3)$

Carbon balance (CB) = (sum of moles of carbon out /sum of moles of carbon in) · 100%

αi represents the number of carbon(s) in the species-i. The steady state results were obtained by taking average of at least 100 mins conversion or selectivity with carbon balance in the range of 100±15%.

Table S1. BET surface area and micropore volume of synthesized catalysts.

**Sample**	**Surface area m^2/g**	**Micropore volume cm^3/g**
Beta-deAl	573	0.175
SnBeta	586	0.177
NaSnBeta	517	0.169
2CeSnBeta	554	0.167
2CeNaSnBeta	526	0.155

Table S2. ICP analysis of synthesized catalysts.

Sample	Element Weight Percentage (%)					Molar Ratios	
	Si	Al	Sn	Na	Ce	Si/Al	Si/Sn
Beta-deAl	43.7	0.07	-	-	-	600	-
SnBeta	43.2	0.07	0.32	-	-	600	575
NaBeta-deAl	-	-	-	0.12	-	-	-
NaSnBeta	-	-	0.31	0.14	-	-	-
2CeSnBeta	-	-	0.26	-	2	-	-

Table S3. ^{1}H NMR quantification of synthesized catalysts.

Catalysts	Peak area (peak ≥ 2 ppm)	Peak area (peak < 2 ppm)
Beta-deAl	3368	1335
SnBeta	2009	889
NaSnBeta	1440	668
NaBeta-deAl	1364	601
2CeSnBeta	1969	941
2CeNaSnBeta	1544	742

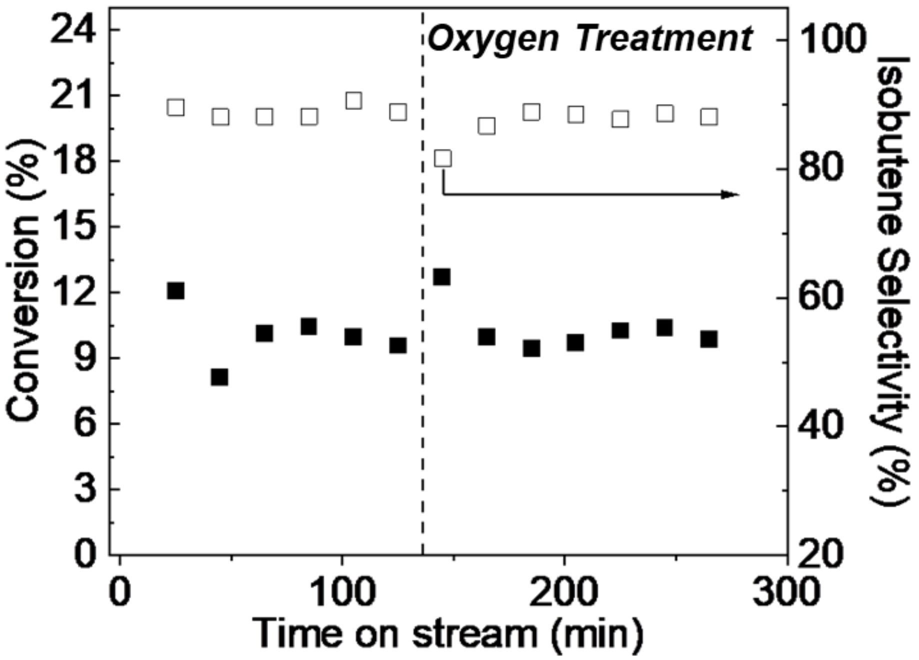

Figure S1. Acetone conversion over 2CeSnNaBeta was evaluated before and after oxygen treatment by flowing 20 SCCM of air for 20 minutes at 673 K. Comparable activity after regeneration indicates the reusability of the catalyst.

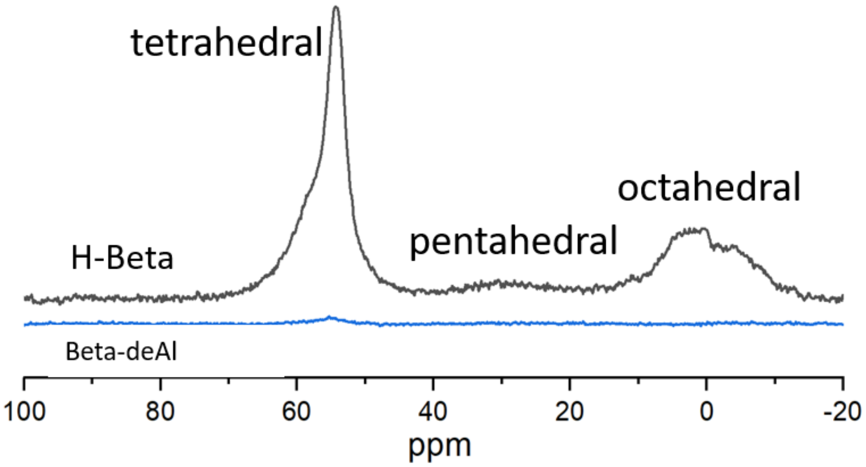

Figure S2. ^{27}Al-SSNMR of H-Beta and dealuminated Beta (Beta-deAl).

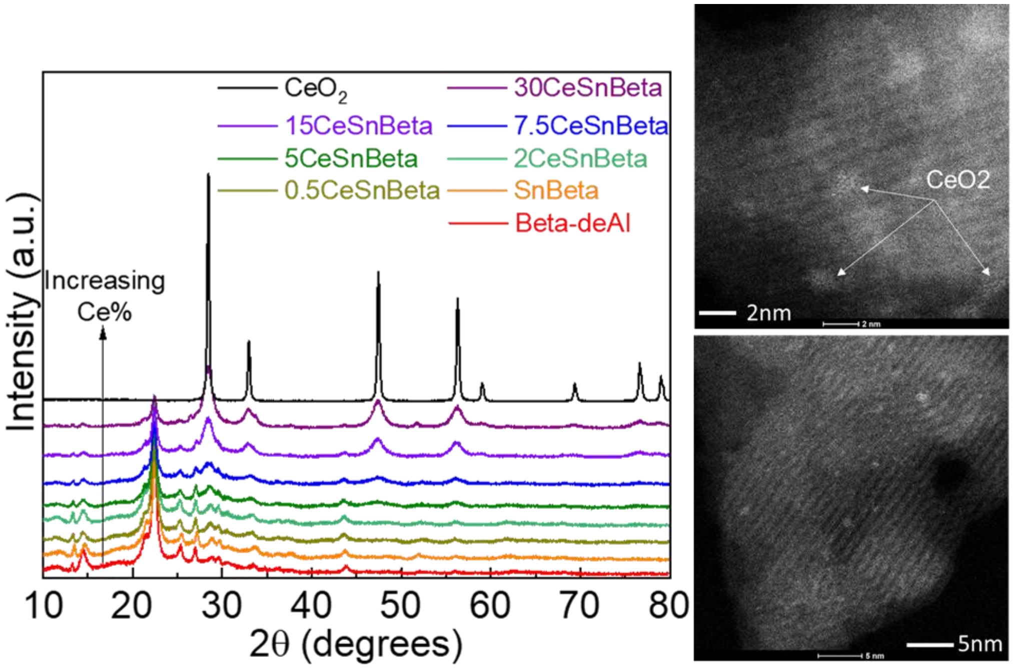

Figure S3. (A) X-ray diffraction (XRD) patterns of synthesized catalysts. (B) Scanning transmission electron microscopy of 2CeSnBeta.

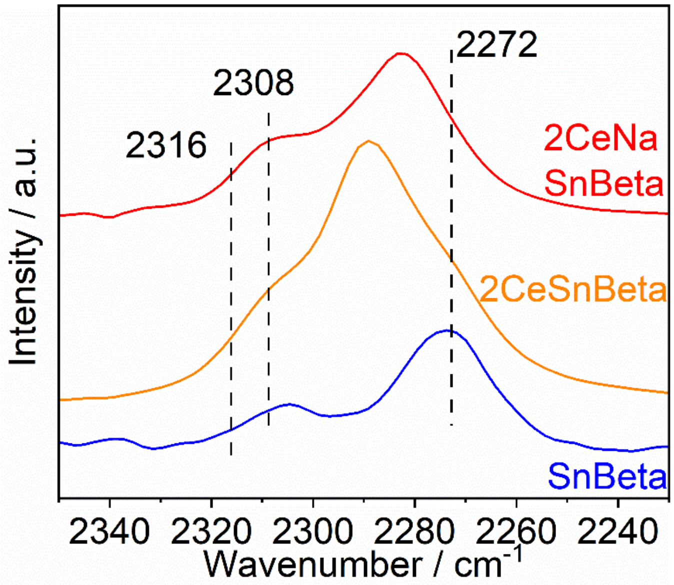

Figure S4. Infrared spectroscopy of $CDCN_3$ adsorbed on SnBeta, 2CeSnBeta, and 2CeNaSnBeta.

We observed CD_3CN adsorbed on closed Sn sites at 2308 cm^{-1} as well as the peak tail at 2316 cm^{-1} (resulted from defect open Sn sites) on all samples.[26] CD_3CN adsorbed on silanol (2272 cm^{-1}) was observed on SnBeta and 2CeSnBeta, while CD3CN adsorption on silanol in 2CeNaSnBeta was barely detectable. The broad peak centered around

2280-2290 cm^{-1} on 2CeSnBeta and 2CenaSnBeta is resulted by CD_3CN adsorbed on CeO_x species.

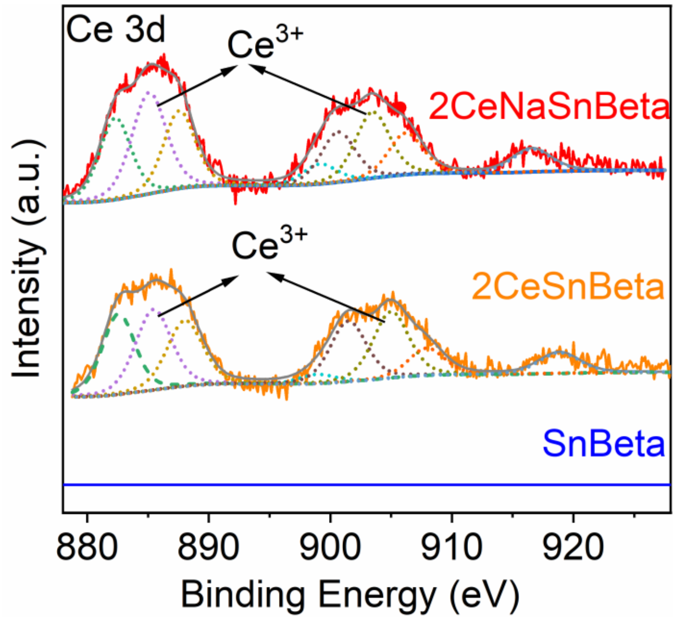

Figure S5. XPS of SnBeta, 2CeSnBeta, and 2CeNaSnBeta. 2CeSnBeta contains 36% Ce^{3+} while 2CeSnNaBeta contains 37% Ce^{3+}.

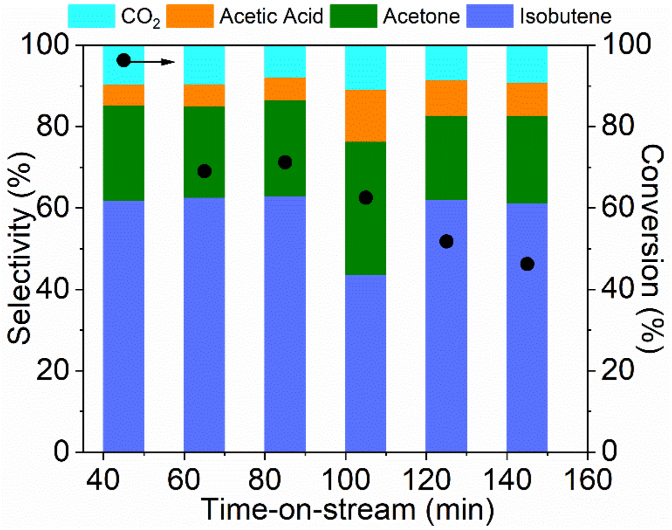

Figure S6. Catalytic performance of mesityl oxide reaction over 2CeNaSnBeta. Reaction conditions: $P_{Mesityl\ oxide}$=0.07 kPa, 673 K; the space velocity=0.25 gMesityl Oxide/gcat/hr.

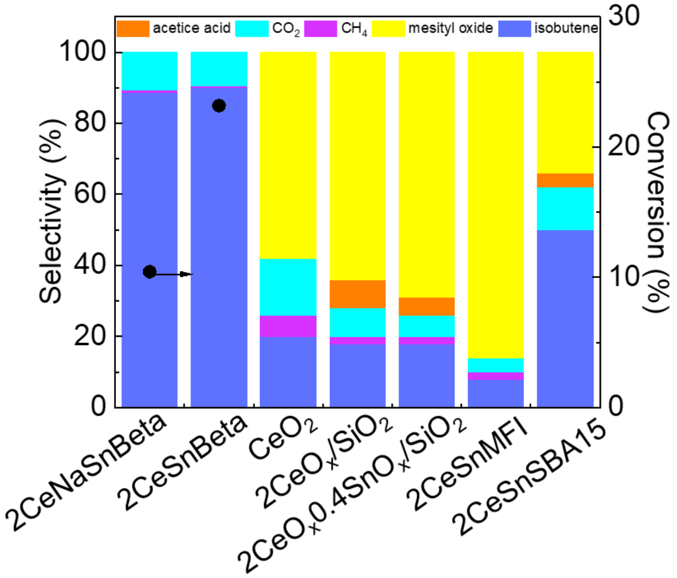

Figure S7. Catalytic performance of 2CeNaSnBeta, 2CeSnBeta, CeO_2, 2%CeO_x/SiO_2, 2%CeO_x0.4%SnO_x/SiO_2, 2CeSnMFI, and 2CeSnSBA15 (the percentage represents weight percentage of Ce or Sn) for acetone conversion. Reaction conditions: $P_{Acetone}$=0.5 kPa, 673 K, averaged 25-100 min time-on-stream results, carbon balance >85%. For 2CeNaSnBeta and 2CeSnBeta, the space velocity=0.23 gAce/gcat/hr; for the rest of the samples, the space velocity was adjusted to measure product distribution at <20% conversion.

References:

(26) Harris, J. W.; Bates, J. S.; Bukowski, B. C.; Greeley, J.; Gounder, R., Opportunities in Catalysis over Metal-Zeotypes Enabled by Descriptions of Active Centers Beyond Their Binding Site. *ACS Catal.* **2020,** *10* (16), 9476-9495.

(30) Osmundsen, C. M.; Holm, M. S.; Dahl, S.; Taarning, E., Tin-Containing Silicates: Structure–Activity Relations. *Proceedings of the Royal Society A: Mathematical, Physical and Engineering Sciences* **2012,** *468* (2143), 2000-2016.

(31) Xu, S.; Jaegers, N. R.; Hu, W.; Kwak, J. H.; Bao, X.; Sun, J.; Wang, Y.; Hu, J. Z., High-Field One-Dimensional and Two-Dimensional ^{27}Al Magic-Angle Spinning Nuclear Magnetic Resonance Study of θ-, δ-, and γ-Al_2O_3 Dominated Aluminum Oxides: Toward Understanding the Al Sites in γ-Al_2O_3. *ACS omega* **2021,** *6* (5), 4090-4099.
